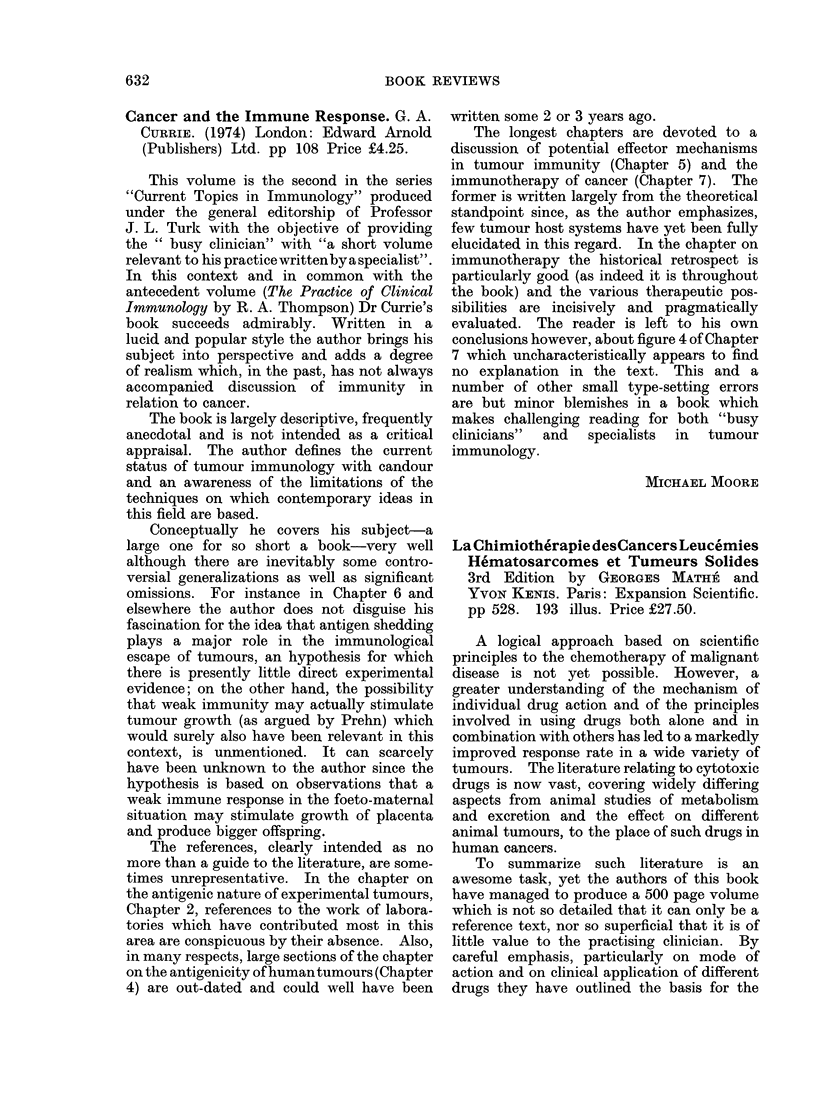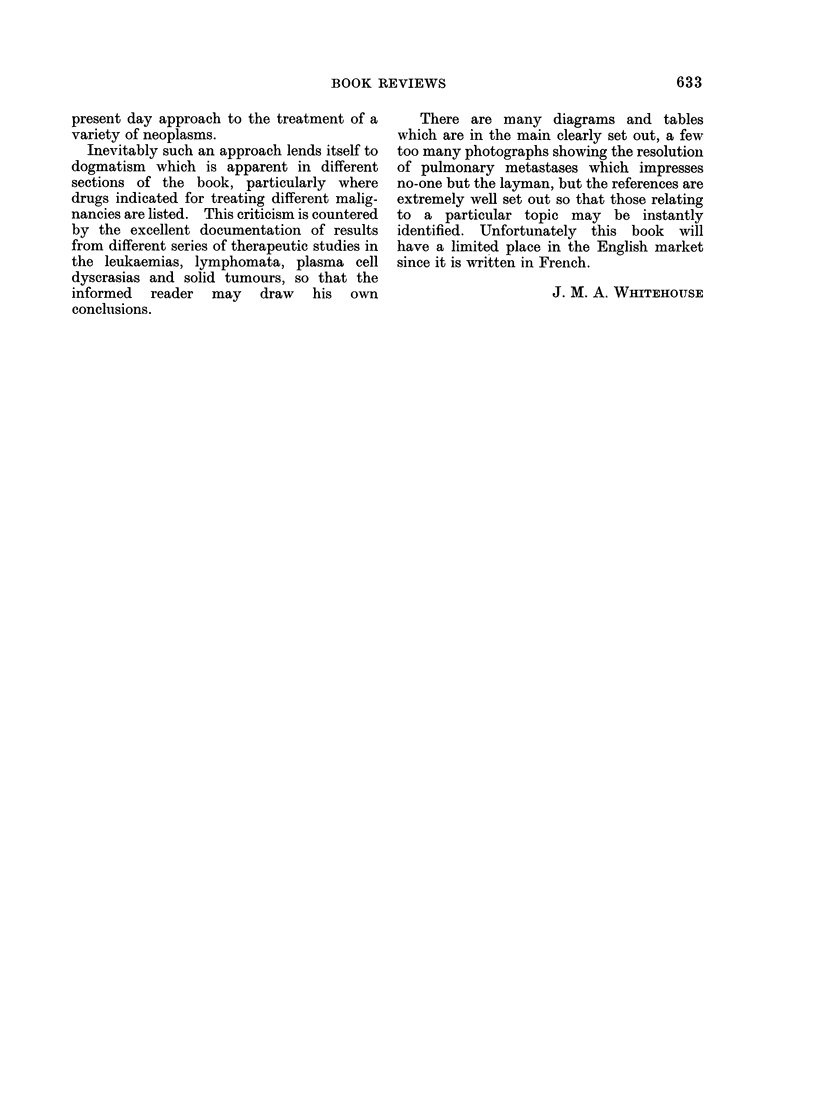# La Chimiothérapie des Cancers Leucémies Hématosarcomes et Tumeurs Solides

**Published:** 1975-11

**Authors:** J. M. A. Whitehouse


**La Chimiothérapie des Cancers Leucémies Hématosarcomes et Tumeurs Solides** 3rd Edition by Georges Mathé and Yvon Kenis. Paris: Expansion Scientific. pp 528. 193 illus. Price £27.50.

A logical approach based on scientific principles to the chemotherapy of malignant disease is not yet possible. However, a greater understanding of the mechanism of individual drug action and of the principles involved in using drugs both alone and in combination with others has led to a markedly improved response rate in a wide variety of tumours. The literature relating to cytotoxic drugs is now vast, covering widely differing aspects from animal studies of metabolism and excretion and the effect on different animal tumours, to the place of such drugs in human cancers.

To summarize such literature is an awesome task, yet the authors of this book have managed to produce a 500 page volume which is not so detailed that it can only be a reference text, nor so superficial that it is of little value to the practising clinician. By careful emphasis, particularly on mode of action and on clinical application of different drugs they have outlined the basis for the present day approach to the treatment of a variety of neoplasms.

Inevitably such an approach lends itself to dogmatism which is apparent in different sections of the book, particularly where drugs indicated for treating different malignancies are listed. This criticism is countered by the excellent documentation of results from different series of therapeutic studies in the leukaemias, lymphomata, plasma cell dyscrasias and solid tumours, so that the informed reader may draw his own conclusions.

There are many diagrams and tables which are in the main clearly set out, a few too many photographs showing the resolution of pulmonary metastases which impresses no-one but the layman, but the references are extremely well set out so that those relating to a particular topic may be instantly identified. Unfortunately this book will have a limited place in the English market since it is written in French.

J. M. A. WHITEHOUSE